# GCCVision: An integrated toolkit for calculating and visualizing parental genome contribution in breeding populations

**DOI:** 10.1016/j.isci.2025.114286

**Published:** 2025-11-28

**Authors:** Enhui Shen, Yifan Yu, Xiaoya Ma, Zhicheng Shen, Yuxuan Ye

**Affiliations:** 1 State Key Laboratory of Rice Biology and Breeding, Key Laboratory of Biology of Crop Pathogens and Insects of Zhejiang Province, Institute of Insect Sciences, Zhejiang University, Hangzhou 310058, China; 2 Zhejiang University Zhongyuan Institute, Zhengzhou 450000, China; 3 Institute of Digital Agriculture, Zhejiang Academy of Agricultural Sciences, Hangzhou, China; 4 The Rural Development Academy, Zhejiang University, Hangzhou 310058, China

**Keywords:** biocomputational method, genomic analysis, plant bioinformatics, plant genetics, techniques in genetics

## Abstract

Tracking parental genome contributions in segregating populations is crucial for accelerating genetic gain in plant breeding. We introduce GCCVision (Genome Contribution Calculator and Visualizer), an integrated bioinformatics toolkit to simplify this process. GCCVision uses an efficient Python-based backend and a user-friendly web-based frontend to analyze Variant Call Format (VCF) files from biparental crosses. The software identifies informative single-nucleotide polymorphisms (SNPs), calculates parental contribution rates, and generates clear, customizable graphical genotype maps where chromosome segments are color-coded by parental origin. By providing clear visualizations of genomic composition, GCCVision assists breeders in selection decisions for backcrossing, F_2_ analysis, quality control of hybrid seeds, and other breeding programs. This streamlined workflow shortens breeding cycles and accelerates the development of improved crop varieties.

## Highlights

• An integrated toolkit for parental genome contribution analysis
• Directly processes standard VCF files from biparental crosses
• Combines a Python backend with an interactive web-based visualizer
• Streamlines selection in breeding programs like backcrossing and F_2_ analysis

## Graphical abstract

**Figure:**
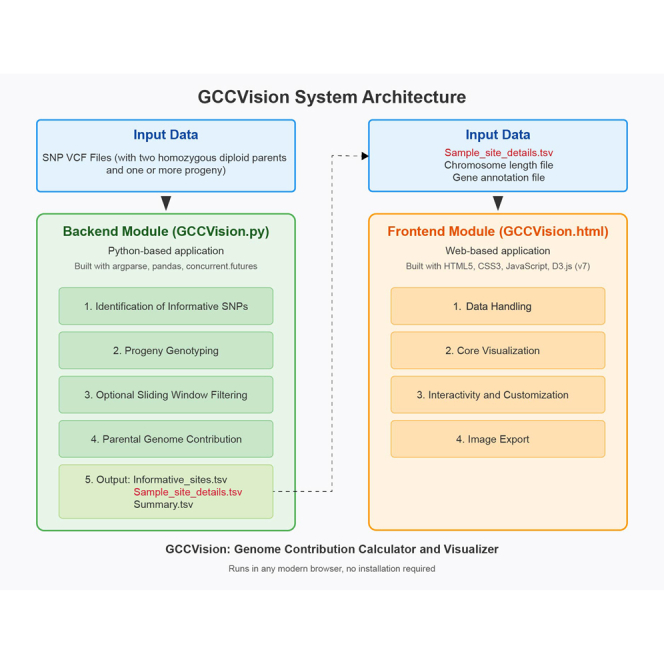

## Introduction

The development of superior plant varieties through breeding is a cornerstone of global agricultural productivity.^1^^,^^2^ Modern breeding has been significantly advanced by molecular tools, making marker-assisted selection an essential strategy for accelerating genetic gain.^3^ Creating segregating populations by crossing two homozygous parents with distinct desirable traits is a foundational strategy central to numerous applications in molecular breeding, from quantitative trait loci (QTLs) mapping to the development of elite cultivars.^4^

This biparental cross approach is particularly critical in marker-assisted backcrossing (MABC). For instance, in transgenic breeding, a desirable gene is often transferred from a donor into an elite, high-yielding recipient line. The primary challenge here is to not only select for the transgene but also rapidly recover the genomic background of the elite parent, thereby minimizing “linkage drag”—the unwanted inheritance of deleterious genes linked to the target locus.^5^^,^^6^ Likewise, in conventional breeding programs for crops like soybean (*Glycine max*), breeders frequently cross two distinct homozygous lines to combine traits such as high yield from one parent and disease resistance from another. In all such cases, success depends on accurately tracking parental genomic segments in subsequent generations (e.g., F_2_ populations) to identify progeny with the desired combination of alleles.^7^^,^^8^

Next-generation sequencing technologies have revolutionized this process by providing an unprecedented volume of high-density single-nucleotide polymorphism (SNP) data, typically stored in the standard Variant Call Format (VCF).^9^^,^^10^ Despite this potential, the large volume of data has created a significant bottleneck between raw genotype generation and its practical application in breeding decisions. To make timely and informed selections, breeders require a tool that provides a clear, quantitative, and visual summary of parental genome recombination in each progeny. This represents a need that is not fully met by the existing bioinformatics landscape.

However, existing bioinformatics tools are often not designed for this specific task. General-purpose VCF manipulation tools (e.g., VCFtools^9^) or variant analysis frameworks (e.g., GATK^11^) are powerful but require complex scripting and multiple steps to get the needed information. Similarly, while genomic data browsers like the Integrative Genomics Viewer (IGV)^12^ are excellent for looking at specific regions, they are not suitable for generating the overview, ideogram-style visualizations needed to compare many samples at once. This workflow gap often forces breeders to perform time-consuming data re-formatting to use graphical genotyping software (e.g., Flapjack^13^), a step that requires bioinformatics skills and can delay selection decisions.^14^^,^^15^ Therefore, there is a distinct need for a specialized tool that streamlines this process by integrating analysis and visualization directly from standard SNP data.

To address this critical gap, we have developed Genome Contribution Calculator and Visualizer (GCCVision), an integrated toolkit that provides an end-to-end solution. It automates the process of identifying informative markers, calculating parental contributions, and generating interactive, publication-quality graphical genotype maps. GCCVision enables breeders and geneticists to transform complex SNP data into intuitive visual summaries, thereby streamlining the selection process in backcrossing, F_2_ analysis, quality control of hybrid seeds, and other breeding programs reliant on biparental populations. This paper describes the architecture, functionality, and utility of GCCVision as a vital tool for modern breeding.

## Results

### Backend data processing and outputs

The GCCVision.py script processes the input VCF file to generate a set of structured, tab-separated output files ready for visualization and further analysis. The initial step identifies all informative SNP markers where the two parents have homozygous but different alleles. Based on these markers, the script then computes key statistics for each progeny. The primary outputs include detailed per-site genotype classifications (as homozygous for parent A, homozygous for parent B, or heterozygous) and a comprehensive summary of parental genome contributions. This summary provides both genome-wide and per-chromosome statistics, quantifying the genetic inheritance from each parent in both absolute and percentage terms. These output files form the foundation for the interactive visualization generated by the frontend module.

### Frontend interactive visualization

The GCCVision.html interface generates interactive chromosome genotype maps from the backend output. The core visualization consists of chromosome ideograms segmented into user-defined bins. Each bin is a stacked bar illustrating the relative proportions of Hom_A (default red), Hom_B (default blue), and Het_AB (default green) sites, providing an intuitive overview of parental genomic blocks and recombination events. The interface is highly interactive, with a control panel for data loading, visual parameter customization, and data export. A key feature is the ability to overlay gene or marker annotations from a user-supplied file, allowing for the immediate visual correlation of specific loci with their underlying parental blocks. This is particularly useful for tracking the introgression of target genes or markers in a breeding program.

### Application case study: Analysis of soybean cultivars

To demonstrate its practical utility, we applied GCCVision to a soybean (*Glycine max*) breeding case, analyzing two sister cultivars, “Suinong 34” (SN34) and “Suinong 36” (SN36), which are sister recombinant inbred lines (RILs) derived from an initial cross between “Suinong 28” (SN28, maternal) and “Heinong 44” (HN44, paternal).^16^ In RILs, the chromosomes are expected to be a mosaic of homozygous blocks from the two parents due to numerous recombination events that occurred in previous generations of self-pollination. The goal was to understand the genomic basis of selection that resulted in two distinct varieties from the same initial cross.

**Figure 1:**
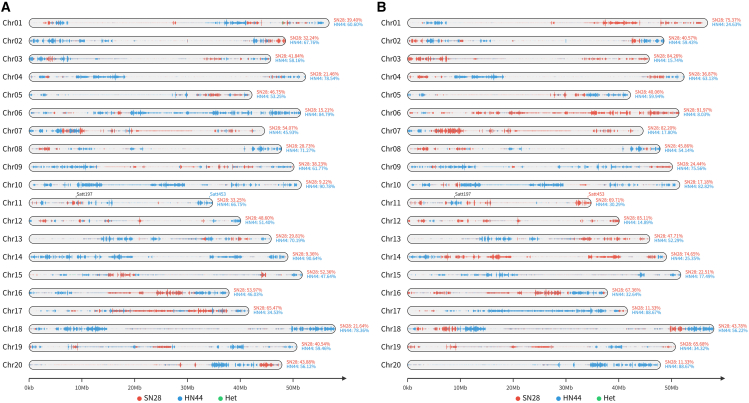
Graphical genotype maps of two soybean sister cultivars generated by GCCVision (A) Graphical genotype map of cultivar “Suinong 34.” (B) Graphical genotype map of “Suinong 36.” Chromosomes are drawn to scale, with bins colored to represent the origin of the genomic segments: red for the maternal parent (SN28: Suinong 28), blue for the paternal parent (HN44: Heinong 44), and green for heterozygous regions (Het).

GCCVision’s quantitative summary quickly revealed that SN34’s genome was predominantly paternal (64.28% from HN44), while SN36 showed a more balanced inheritance (48.75% from HN44). The graphical genotype map highlighted a major genomic divergence on chromosome 6: SN34 inherited a large paternal block (84.79% from HN44) (Figure 1A), whereas SN36 almost entirely retained the maternal haplotype (91.97% from SN28) (Figure 1B). This visualization pinpoints a key region likely responsible for phenotypic differences. Furthermore, we tracked two SSR (Simple Sequence Repeats) loci on chromosome 11: Satt197 (monomorphic between SN28 and HN44) and Satt453 (polymorphic between SN28 and HN44). As expected, the visualization showed that Satt197 was identical in both cultivars, while for Satt453, SN34 inherited the paternal allele and SN36 inherited the maternal allele.

### Application case study: Assessing the purity of maize hybrid seeds

Beyond analyzing segregating populations, GCCVision is also highly effective for quality control in hybrid seed production. The genetic purity of F_1_ hybrid seeds is crucial for ensuring uniform and superior crop performance. An ideal F_1_ hybrid, resulting from a cross between two distinct homozygous inbred lines, is expected to be heterozygous genome-wide and inherit approximately 50% of its genome from each parent.

To showcase this application, we used GCCVision to assess the purity of three commercial maize (*Zea mays*) hybrids: JingKe968 (parents: Jing724 and Jing92), ZhengDan958 (parents: Chang7-2 and Zheng58), and YuFeng303 (parents: CT3354 and CT1669). Our analysis revealed that all three hybrids exhibit a parental genome contribution ratio very close to the expected 1:1 (Table 1). Genome-wide, the parental contributions were 50.14% and 49.86% for JingKe968, 50.45% and 49.55% for ZhengDan958, and 50.04% and 49.96% for YuFeng303. This balanced inheritance was consistent across all individual chromosomes for each hybrid, with only minor fluctuations, confirming the absence of large-scale genomic aberrations or contamination. These results confirm the high genetic purity of the tested hybrid samples and demonstrate that GCCVision provides a rapid and reliable method for quality control in commercial seed production.

**Table 1:** Parental genome contributions in three commercial maize hybrids

Sample	Chromosome	Parent_A_Contribution(%)	Parent_B_Contribution(%)
YuFeng303	genome-wide	50.0431	49.9569
	Chr01	49.9985	50.0015
	Chr02	49.9810	50.0190
	Chr03	50.0473	49.9527
	Chr04	50.1718	49.8282
	Chr05	50.0020	49.9980
	Chr06	50.2313	49.7687
	Chr07	49.9027	50.0973
	Chr08	49.9947	50.0053
	Chr09	50.2468	49.7532
	Chr10	49.8432	50.1568
ZhengDan958	genome-wide	50.4523	49.5477
	Chr01	50.4705	49.5295
	Chr02	50.5150	49.4850
	Chr03	50.4298	49.5702
	Chr04	50.3779	49.6221
	Chr05	50.3833	49.6167
	Chr06	50.4968	49.5032
	Chr07	50.3165	49.6835
	Chr08	50.5935	49.4065
	Chr09	50.4674	49.5326
	Chr10	50.4789	49.5211
JingKe968	genome-wide	50.1374	49.8626
	Chr01	50.1867	49.8133
	Chr02	50.0584	49.9416
	Chr03	50.0683	49.9317
	Chr04	50.2445	49.7555
	Chr05	50.1142	49.8858
	Chr06	50.1409	49.8591
	Chr07	50.0910	49.9090
	Chr08	50.2069	49.7931
	Chr09	50.0940	49.9060
	Chr10	50.1017	49.8983

## Discussion

The analysis of parental genome contribution from large-scale genotyping data presents a common challenge in breeding programs: how to efficiently translate vast, complex datasets into clear, interpretable results for decision-making. GCCVision is designed to address this challenge by offering an integrated and streamlined workflow. Its main contribution is the direct linkage of quantitative analysis with interactive visualization, starting from the standard VCF file. This approach lowers the barrier to entry for users who may not have extensive bioinformatics scripting skills, allowing them to rapidly assess introgression patterns across entire populations.

GCCVision is designed to fill a specific gap in the bioinformatics toolkit for breeders (Table 2). While command-line tools like VCFtools^9^ and GATK^11^ are indispensable for their core functions, they are not designed for this specific analytical and visualization task. Similarly, viewers like IGV^12^ are optimized for detailed region inspection not summary ideograms, while other tools, such as Flapjack,^13^ MG2C,^17^ or R qtl2,^18^ require data re-formatting or programming expertise, respectively. GCCVision bridges this gap by offering a streamlined, end-to-end workflow: it takes standard SNP data as direct input, performs the contribution analysis, and outputs an interactive visualization, thus making this analysis more accessible, especially for researchers with limited bioinformatics experience.

**Table 2:** Comparison of GCCVision with other relevant bioinformatics tools

Feature	GCCVision	VCFtools	IGV	Flapjack	R (qtl2)
Primary design goal	integrated parental contribution analysis	VCF manipulation and filtering	interactive genome browsing	graphical genotype visualization	QTL genetics
Direct VCF input	yes	yes	yes	no	no
Calculates parental contribution %	yes	no	no	no	yes
Generates graphical genotype maps	yes	no	no	yes	yes
Interactive visualization	yes	no	yes	yes	no
Requires programming	no	yes	no	no	yes

The utility of GCCVision workflow is significant across several breeding scenarios. In MABC, the tool offers a direct way to visualize the recovery of the recurrent parent genome while simultaneously tracking the introgression of one or more donor segments, helping to accelerate recovery and select against linkage drag.^10^ For the analysis of segregating populations, such as the F_2_ populations used in our soybean case study, GCCVision facilitates the rapid identification of individuals with desirable recombination events, which is crucial for QTL mapping and the selection of parents for the next breeding cycle.^19^

The workflow can be further optimized using the optional inputs. For instance, when analyzing a large population, the informative SNPs can be identified once and saved. Subsequent analyses on subsets of progeny can then use the --informative-sites option to bypass the time-consuming VCF parsing step. Similarly, providing a gene annotation file allows users to overlay key loci (e.g., a disease resistance gene) on the genotype map, providing immediate visual confirmation of a successful introgression.

Furthermore, as demonstrated in our maize case study, GCCVision can serve as an effective quality control tool to confirm parentage, assess the genetic purity of hybrid lines, and identify potential outcrosses or sample-handling errors.

Looking forward, the current version of GCCVision is intentionally focused on a common and important scenario in breeding: analyzing diploid populations derived from two homozygous parents. This focused design ensures the tool’s performance and ease of use for these specific applications. Future development could aim to broaden its applicability by incorporating support for polyploid species and more complex crosses. Another valuable extension would be a comparative visualization mode, allowing for the side-by-side display of multiple individuals on a single canvas to more easily contrast recombination patterns within a family. Such enhancements would further increase the utility of GCCVision for the genetics and breeding community.

In summary, GCCVision provides a specialized and user-friendly toolkit that addresses a key challenge in modern breeding: the analysis and visualization of parental genome contribution. By efficiently converting SNP data into clear and interactive graphical genotypes, it enables breeders and geneticists to make more informed selection decisions. This tool can help shorten breeding cycles and accelerate the development of improved crop varieties. The current version is focused on diploid biparental populations and provides a solid base for future development to support more complex genetic systems, making it a useful tool for the plant science community.

### Limitations of the study

The current version of GCCVision is specifically designed for analyzing diploid organisms derived from crosses between two homozygous parents. Its applicability is therefore limited in the context of polyploid species or more complex breeding schemes, such as multi-parent populations. The accuracy of the output is highly dependent on the quality of the input VCF file, as the tool does not perform upstream variant filtering and assumes that standard quality control has already been applied. While the optional sliding-window filter can mitigate the impact of sporadic genotyping errors, it is not designed to correct for systematic biases or low-quality data. Finally, the visualization is currently optimized for analyzing individual progeny, and a comparative mode for displaying multiple individuals side-by-side has been identified as a valuable future extension.

## Resource availability

### Lead contact

Further information and requests for resources should be directed to and will be fulfilled by the lead contact, Yuxuan Ye (yeyuxuan@zju.edu.cn).

### Materials availability

This study did not generate new unique reagents.

### Data and code availability

• Publicly available whole-genome resequencing data used in this study were obtained from the NCBI Sequence Read Archive under BioProject accessions PRJNA681974, PRJNA1202942, and PRJNA1170466. These accession numbers are listed in the key resources table.
• The GCCVision software, including the Python script (GCCVision.py), frontend files (GCCVision.html and its dependencies), example datasets, and documentation, is freely available on GitHub at https://github.com/nhyyx37/GCCVision.
• Any additional information required to reanalyze the data reported in this paper is available from the lead contact upon request.

## Acknowledgments

This work was supported by the 10.13039/501100001809National Natural Science Foundation of China (32201774) and the 10.13039/100022963Key Research and Development Program of Zhejiang Province (2023C02033).

## Author contributions

Y.X.Y. conceived the original idea, supervised the entire work, and drafted the manuscript. E.H.S. designed the overall workflow and developed GCCVision algorithm. Y.F.Y. and X.Y.M. performed the case study analyses and helped validate the tool using real-world data. Z.C.S. provided valuable suggestions during the development phase. All authors participated in reviewing and improving the manuscript.

## Declaration of interests

The authors declare no competing interests.

## STAR★Methods

### Key resources table

**Table:** 

REAGENT or RESOURCE	SOURCE	IDENTIFIER
**Deposited data**		

Whole-genome resequencing data for soybean and maize case studies	NCBI Sequence Read Archive	BioProject: PRJNA681974, PRJNA1202942, PRJNA1170466

**Software and algorithms**		

GCCVision	This paper	https://github.com/nhyyx37/GCCVision
Python 3	Python Software Foundation	https://www.python.org
pandas	The pandas development team	https://pandas.pydata.org
D3.js (v7)	Mike Bostock	https://d3js.org

### Experimental model and study participant details

#### Soybean (*Glycine max*)

The soybean analysis was conducted on two sister recombinant inbred lines (RILs), ‘Suinong 34’ (SN34) and ‘Suinong 36’ (SN36). These lines were derived from an initial cross between the maternal parent ‘Suinong 28’ (SN28) and the paternal parent ‘Heinong 44’ (HN44).

#### Maize (*Zea mays*)

The maize analysis was conducted on three commercial F_1_ hybrid cultivars to assess their genetic purity. The hybrids and their respective parental lines were: JingKe968 (parents: Jing724 and Jing92), ZhengDan958 (parents: Chang7-2 and Zheng58), and YuFeng303 (parents: CT3354 and CT1669).

### Method details

#### System architecture

GCCVision consists of two interconnected components. Backend Module (GCCVision.py): a command-line script implemented in Python 3. It leverages standard libraries such as argparse, pandas, and concurrent.futures for argument parsing, efficient data manipulation, and parallel processing, respectively. Frontend Module (GCCVision.html): a self-contained web application built with HTML5, CSS3, and JavaScript. It utilizes the D3.js library (v7) for dynamic data visualization and supports client-side file saving and image export. The frontend runs in any modern web browser without requiring installation.

#### Input data

GCCVision requires a multi-sample VCF file (plain text or gzipped) containing genotype information for two homozygous diploid parents and one or more progeny. Additionally, the visualization module requires a tab-separated file defining chromosome names and their physical lengths. Optional inputs include a pre-computed informative sites file and a gene annotation file for overlaying markers. The VCF file is a standard output of many variant calling pipelines; an example workflow using GATK for generating this file from raw sequencing reads is provided in the Data S1.

#### Backend processing module (GCCVision.py)

The backend script executes a sequential pipeline controlled by command-line parameters. To optimize performance, it processes chromosomes in parallel, with the level of parallelism controlled by the --threads parameter. The key processing steps are: 1. Identification of Informative SNPs: The script parses the VCF file to identify sites where Parent A and Parent B are homozygous for different alleles. 2. Progeny Genotyping: At each informative SNP, progeny genotypes are classified as homozygous for the Parent A allele (Hom_A), homozygous for the Parent B allele (Hom_B), or heterozygous (Het_AB). 3. Optional Sliding Window Filtering: An optional filtering algorithm can be applied to reduce the impact of potential genotyping errors. The --filter-window-size (-w, default: 5) parameter defines the number of neighboring SNPs to consider for correcting an inconsistent central SNP. The --filter-times (-f, default: 0) parameter specifies the number of filtering iterations. A value of 0 disables the filter.

The main command-line parameters for the backend script GCCVision.py include: --vcf (-v) for the input VCF file (required); --parent-a (-a) and --parent-b (-b) for the sample names of Parent A and Parent B (required unless an informative sites file is provided); --samples (-s) for a list of progeny sample names (required); and --output-prefix (-o) for the prefix for all output files (required). An optional --informative-sites (-i) parameter can be used to provide a pre-existing informative sites file.

#### Frontend visualization module (GCCVision.html)

The frontend module provides a graphical user interface for data exploration, loading the chromosome length and site detail files generated by the backend. The main features include: Data Handling: User-friendly inputs for chromosome, site, and optional gene data files, with an option to load a pre-packaged sample dataset; Core Visualization: Chromosomes are drawn to scale as graphical genotypes, with genomic regions color-coded by parental origin (Hom_A, Hom_B, Het_AB) using a stacked histogram. Bin size is user-configurable for multi-resolution analysis; Interactivity and Customization: Users can toggle the visibility of different genotype regions, adjust display parameters (e.g., dimensions, colors, fonts), and overlay gene markers from an optional annotation file; Export: The visualization can be exported as high-resolution PNG, JPEG, or lossless SVG files.

### Quantification and statistical analysis

The backend script calculates parental genome contribution rates by tallying the total number of alleles from Parent A and Parent B across all informative SNP sites. A site homozygous for the Parent A allele (Hom_A) contributes two ‘A’ alleles, a site homozygous for the Parent B allele (Hom_B) contributes two ‘B’ alleles, and a heterozygous site (Het_AB) contributes one allele from each parent. These calculations provide both genome-wide and per-chromosome statistics, quantifying the genetic inheritance from each parent in both absolute and percentage terms. The results are summarized in tab-separated output files for visualization and further analysis.

## Supplemental information

Data S1. GATK-based variant calling pipeline

## References

[1] Godfray H.C.J., Beddington J.R., Crute I.R., Haddad L., Lawrence D., Muir J.F., Pretty J., Robinson S., Thomas S.M., Toulmin C. (2010). Food security: the challenge of feeding 9 billion people. Science.

[2] Tester M., Langridge P. (2010). Breeding technologies to increase crop production in a changing world. Science.

[3] Collard B.C.Y., Mackill D.J. (2008). Marker-assisted selection: an approach for precision plant breeding in the twenty-first century. Philos. Trans. R. Soc. Lond. B Biol. Sci..

[4] Moose S.P., Mumm R.H. (2008). Molecular plant breeding as the foundation for 21st century crop improvement. Plant Physiol..

[5] Hospital F. (2005). Selection in backcross programmes. Philos. Trans. R. Soc. Lond. B Biol. Sci..

[6] Frisch M., Melchinger A.E. (2005). Selection theory for marker-assisted backcrossing. Genetics.

[7] Lander E.S., Botstein D. (1989). Mapping mendelian factors underlying quantitative traits using RFLP linkage maps. Genetics.

[8] He J., Zhao X., Laroche A., Lu Z.X., Liu H., Li Z. (2014). Genotyping-by-sequencing (GBS), an ultimate marker-assisted selection (MAS) tool to accelerate plant breeding. Front. Plant Sci..

[9] Danecek P., Auton A., Abecasis G., Albers C.A., Banks E., DePristo M.A., Handsaker R.E., Lunter G., Marth G.T., Sherry S.T. (2011). The variant call format and VCFtools. Bioinformatics.

[10] Rasheed A., Hao Y., Xia X., Khan A., Xu Y., Varshney R.K., He Z. (2017). Crop breeding chips and genotyping platforms: progress, challenges, and perspectives. Mol. Plant.

[11] McKenna A., Hanna M., Banks E., Sivachenko A., Cibulskis K., Kernytsky A., Garimella K., Altshuler D., Gabriel S., Daly M., DePristo M.A. (2010). The Genome Analysis Toolkit: a MapReduce framework for analyzing next-generation DNA sequencing data. Genome Res..

[12] Robinson J.T., Thorvaldsdóttir H., Winckler W., Guttman M., Lander E.S., Getz G., Mesirov J.P. (2011). Integrative genomics viewer. Nat. Biotechnol..

[13] Milne I., Shaw P., Stephen G., Bayer M., Cardle L., Thomas W.T.B., Flavell A.J., Marshall D. (2010). Flapjack—graphical genotype visualization. Bioinformatics.

[14] Varshney R.K., Ribaut J.M., Buckler E.S., Tuberosa R., Rafalski J.A., Langridge P. (2012). Can genomics boost productivity of orphan crops?. Nat. Biotechnol..

[15] Cobb J.N., Declerck G., Greenberg A., Clark R., McCouch S. (2013). Next-generation phenotyping: requirements and strategies for enhancing our understanding of genotype–phenotype relationships and its relevance to crop improvement. Theor. Appl. Genet..

[16] Li Y.-H., Qin C., Wang L., Jiao C., Hong H., Tian Y., Li Y., Xing G., Wang J., Gu Y. (2023). Genome-wide signatures of the geographic expansion and breeding of soybean. Sci. China Life Sci..

[17] Chao J., Li Z., Sun Y., Aluko O.O., Wu X., Wang Q., Liu G. (2021). MG2C: A user-friendly online tool for drawing genetic maps. Mol. Hortic..

[18] Broman K.W., Gatti D.M., Simecek P., Furlotte N.A., Prins P., Sen Ś., Yandell B.S., Churchill G.A. (2019). R/qtl2: software for mapping quantitative trait loci with high-dimensional data and multiparent populations. Genetics.

[19] Poland J.A., Rife T.W. (2012). Genotyping-by-Sequencing for Plant Breeding and Genetics. Plant Genome.

