# Supplementary material for: GCCVision: An integrated toolkit for calculating and visualizing parental genome contribution in breeding populations
Source: iScience. 2025 Nov 28;29(1):114286. doi: 10.1016/j.isci.2025.114286 (PMC12757579; doi:10.1016/j.isci.2025.114286)
Supplement: Data S1. GATK-based variant calling pipeline [file mmc1.pdf]

iScience, Volume *29*

## Supplemental information

# GCCVision: An integrated toolkit for calculating and visualizing parental genome contribution in breeding populations

**Enhui Shen, Yifan Yu, Xiaoya Ma, Zhicheng Shen, and Yuxuan Ye**

**Supplemental Data S1:**

# GATK-Based Variant Calling Pipeline

This script outlines the main steps to identify SNPs from raw sequencing reads using BWA and GATK.

## Step 1: Reference Genome Indexing

**Goal:** Create index files for the reference genome. This only needs to be done once.

```
## Create BWA index for alignment
bwa index genome.fasta

## Create a sequence dictionary for GATK
gatk CreateSequenceDictionary -R genome.fasta

## Create a FASTA index for samtools
samtools faidx genome.fasta
```

## Step 2: Read Alignment and BAM Processing (Per Sample)

**Goal:** To align the raw sequencing reads for each sample to the reference genome and then sort, mark duplicates, and index the resulting alignment file (BAM). This entire section should be run for each sample.

```
## The 'sample' variable should be set for each sample you process
sample="sample_name"

## 1. Align reads and convert to BAM format
# The -R flag adds a required Read Group header, which is essential for GATK.
bwa mem -t 40 -R "@RG\tID:${sample}\tPL:ILLUMINA\tLB:$sample\tSM:$sample" \
    genome.fasta ${sample}.1.fastq.gz ${sample}.2.fastq.gz | samtools view -Sb -> ${sample}.bam

## 2. Sort the BAM file by genomic position
samtools sort -@ 8 -o ${sample}.sorted.bam ${sample}.bam

## 3. Mark and remove PCR duplicates
gatk MarkDuplicates -I ${sample}.sorted.bam -O ${sample}.sorted.markdup.bam -M ${sample}.markdup_metrics.txt

## 4. Index the final BAM file for fast access
samtools index ${sample}.sorted.markdup.bam
```

**Step 3: Per-Sample Variant Calling with HaplotypeCaller**

Goal: Call variants for each sample and then split the result by chromosome to allow for parallel processing later.

```
## Call variants in GVCF mode for the whole sample
gatk HaplotypeCaller -R genome.fasta -I ${sample}.sorted.markdup.bam -O ${sample}.raw.g.vcf.gz --emit-ref-confidence GVCF

## Split the GVCF by chromosome (assumes a file 'genome.chr.list' exists)
for chr in $(cat genome.chr.list)
do
    gatk SelectVariants -V ${sample}.raw.g.vcf.gz -O ${sample}.raw.${chr}.vcf.gz -L ${chr}
done
```

**Step 4: Consolidate GVCFs and Joint-Genotype (Per Chromosome)**

**Goal:** Combine GVCFs from all samples and call genotypes together, one chromosome at a time.

```
## This loop runs once for each chromosome
for chr in $(cat genome.chr.list)
do
    # Create a list of GVCFs for the chromosome
    ls *${chr}.vcf.gz > ${chr}.gvcf.raw.list
    # Import all samples into a GenomicsDB
    gatk GenomicsDBImport -R genome.fasta -V ${chr}.gvcf.raw.list --genomicsdb-workspace-path ${chr}_database -L ${chr}
    # Jointly call genotypes from the database
    gatk GenotypeGVCFs -R genome.fasta -V gendb://${chr}_database -O ${chr}.variants.vcf.gz
done
```

**5. Step 5: Variant Filtering and SNP Selection (Per Chromosome)**

**Goal:** Apply filters to remove low-quality variant calls and keep only high-quality SNPs.

```
## This loop runs once for each chromosome
for chr in $(cat genome.chr.list)
do
    # Apply filters to flag low-quality variants (e.g., based on FS, QD, DP scores)
    gatk VariantFiltration -R genome.fasta -V ${chr}.variants.vcf.gz -O ${chr}.filtered.vcf.gz \
        --cluster-window-size 35 --cluster-size 3 \
        --filter-name "FS_filter" -filter "FS > 30.0" \
        --filter-name "QD_filter" -filter "QD < 2.0" \
        --filter-name "DP_filter" -filter "DP < 10"
```

```
    # Select only the SNPs that passed the filters
    gatk SelectVariants -V ${chr}.filtered.vcf.gz -O ${chr}.final.snps.vcf.gz \
        --select-type-to-include SNP --exclude-filtered
done
```

**Step 6: Final VCF Merging and Cleanup**

Goal: Combine the per-chromosome VCFs into one final, genome-wide file.

```
## List all final chromosome VCFs
ls *.final.snps.vcf.gz > final.snps.vcf.list

## Merge into a single genome-wide VCF
gatk MergeVcfs -I final.snps.vcf.list -O final.snps.vcf.gz

## (Optional) Extract sample names from the final VCF
bcftools query -l final.snps.vcf.gz > samples.txt
```
